# Comparing field and lab quantitative stable isotope probing for nitrogen assimilation in soil microbes

**DOI:** 10.1128/aem.01849-24

**Published:** 2025-01-16

**Authors:** Kinsey Reed, Chansotheary Dang, Jeth Walkup, Alicia Purcell, Bruce Hungate, Ember Morrissey

**Affiliations:** 1 Division of Plant and Soil Sciences, West Virginia University 5631 , Morgantown, West Virginia, USA; 2 Department of Biological Sciences, Texas Tech University 124573 , Lubbock, Texas, USA; 3 Center for Ecosystem Science and Society, Northern Arizona University 519337 , Flagstaff, Arizona, USA; 4 Department of Biological Sciences, Northern Arizona University 222339 , Flagstaff, Arizona, USA; University of Delaware, Lewes, Delaware, USA

**Keywords:** quantitative methods, agroecosystems, soil microbiology, nitrogen assimilation, nitrogen immobilization, rhizosphere, microbial communities, molecular methods, maize microbiome

## Abstract

Soil microbial communities play crucial roles in nutrient cycling and can help retain nitrogen in agricultural soils. Quantitative stable isotope probing (qSIP) is a useful method for investigating taxon-specific microbial growth and utilization of specific nutrients, such as nitrogen (N). Typically, qSIP is performed in a highly controlled lab setting, so the field relevance of lab qSIP studies remains unknown. We conducted and compared tandem lab and field qSIP to quantify the assimilation of ^15^N by maize-associated soil prokaryotic communities at two agricultural sites. Here, we show that field qSIP with ^15^N can be used to measure taxon-specific microbial N assimilation. Relative ^15^N assimilation rates were generally lower in the field, and the magnitude of this difference varied by site. Rates differed by method (lab vs field) for 19% of the top N assimilating genera. The field and lab measures were more comparable when relative assimilation rates were weighted by relative abundance to estimate the proportion of N assimilated by each genus with only ~10% of taxa differing by method. Of those that differed, the taxa consistently higher in the lab were inclined to have opportunistic lifestyle strategies, whereas those higher in the field had niches reliant on plant roots or in-tact soil structure (biofilms, mycelia). This study demonstrates that ^15^N-qSIP can be successfully performed using field-incubated soils to identify microbial allies in N retention and highlights the strengths and limitations of field and lab qSIP approaches.

**IMPORTANCE:**

Soil microbes are responsible for critical biogeochemical processes in natural and agricultural ecosystems. Despite their importance, the functional traits of most soil organisms remain woefully under-characterized, limiting our ability to understand how microbial populations influence the transformation of elements such as nitrogen (N) in soil. Quantitative stable isotope probing (qSIP) is a powerful tool to measure the traits of individual taxa. This method has rarely been applied in the field or with ^15^N to measure nitrogen assimilation. In this study, we measured genus-specific microbial nitrogen assimilation in two agricultural soils and compared field and lab ^15^N qSIP methods. Our results identify taxa important for nitrogen assimilation in agricultural soils, shed light on the field relevance of lab qSIP studies, and provide guidance for the future application of qSIP to measure microbial traits in the field.

## INTRODUCTION

Microbes play a vital role in soil ecosystem functions, especially nitrogen (N) cycling, as they immobilize, mineralize, oxidize, and reduce N, determining its accessibility to plants (1, 2). These processes—and the microbes that drive them—are likely sensitive to environmental change, sensitivities we need to understand (3). Historically, our ability to tie microbial community composition to ecosystem function has been limited.

Recent methodological advances help decipher the overwhelmingly complex influence of microbial life on biogeochemical processes (4). Quantitative stable isotope probing (qSIP) is a powerful technique that can help identify which microorganisms are responsible for specific ecosystem processes, such as carbon (C) and N assimilation, by tracing the incorporation of isotopically labeled, or “heavy,” substrates into DNA (5–8). Often qSIP is conducted on microbes living under varying experimental conditions, for example, temperature, moisture, or fertilization, to better predict how microbes might respond to anthropogenic influences in the wild (9–14). This technique is most often performed using ^18^O water to quantify taxon-specific growth rates or using ^13^C substrates to quantify C assimilation rates. With the development of methods for ^15^N qSIP (15, 16), and the importance of microbes for soil N cycling, qSIP is starting to be used to assess microbial N assimilation.

Nitrogen is the most common limiting nutrient to crop growth in temperate soil and must be supplemented in many agricultural systems to maintain productivity. Soil microbes transform nitrogenous compounds, and N is readily lost from soil through processes performed or influenced by microbes (2). Yet, microbes can also help soils retain N by assimilating it into their biomass, promoting soil N retention through immobilization (17). This is of particular importance in agricultural soil, where expensive fertilizer-N is regularly applied for production. It is estimated that only 40%–53% of applied N globally is taken up by crops as intended (18). Leached N ends up in waterways, resulting in pollution and eutrophication (19). When soil microbes assimilate N into their biomass, it is more likely to be retained in the soil and gradually released to crops over time as those microbes live and die (20–22), estimating that the contribution of bacterial and fungal residue N to soil organic N is between 27% and 100% (22). Implementing agricultural management practices that result in sustained abundance and growth of microbes that immobilize fertilizer N—whether due to their traits, cell or population size, or some combination thereof—could reduce soil N loss (23–26).

It is possible to quantify N assimilation rates of individual microbes using qSIP. Microbial community response to N fertilization is often assessed by DNA sequencing, which cannot distinguish active microbes from those that are dormant or recently dead (27, 28). In the context of N assimilation, active populations are most relevant and responsive to fertilization (29). Nevertheless, to the best of our knowledge, only a few studies have used ^15^N thus far to measure microbial N assimilation (9, 10, 15, 30–33), and even fewer have focused on row-crop agricultural soils (33, 34).

One concern regarding the use of qSIP to explore microbial responses to environmental change is that the rates observed in the lab may not be field-relevant. This concern is shared for most biogeochemical analyses, and often there is disagreement between lab and field studies, particularly for N-cycling rates (35–37). Lab measurements are often used because they are more precise, more practical, and less expensive. However, they are more likely to introduce unintended artifacts from plant removal and soil processing which may include sieving, homogenization, and drying or rewetting.

It is important to capture field-relevant processes in agricultural systems that include dynamic plant-soil interactions. Plant-soil-microbial feedback loops drive nutrient cycling in agricultural settings (38, 39) and shape microbial community composition and activity in the soil surrounding their roots. The rhizosphere, defined as the soil immediately surrounding plant roots and their fungal symbionts, is a hot spot of microbial activity, nutrient cycling, and networking (40). Plant roots and root secretions support a complex economy with fluctuating proportions of plant mutualists and pathogens, as well as microbes that capitalize on the resource-rich environment without directly impacting the plant. Accordingly, the removal of plant roots prior to lab qSIP measurements may disrupt the composition and functioning of rhizosphere microbes.

Understanding the impact of laboratory artifacts on qSIP measurements is necessary to determine if microbial functions measured in the lab reflect their activities in the wild. Although several qSIP experiments have been conducted in the field (11, 13, 14, 32, 41), no past works have directly compared field and lab measures of microbial activity via qSIP. As such, the extent to which artifacts (from sieving, root removal, etc.) influence lab measurements remains unknown. In addition, to our knowledge, no prior field qSIP studies have examined nitrogen assimilation with ^15^N or focused on agricultural soil.

This work aimed to (i) determine whether and how genus-specific N assimilation as assessed via qSIP differs between lab and field measurements and (ii) identify soil prokaryotes important for N assimilation in the rhizosphere of maize (*Zea mays*). We hypothesized that lab conditions could inflate rates of N assimilation relative to field conditions due to more optimal growth conditions and lowered competition for N from plants. We also predicted the function and abundance of root-associated microbial groups would differ between the field and lab measurements, given the root removal and soil disturbance that occurs during the lab procedure. To address the aims and hypotheses, we performed qSIP with ^15^N in the field and the lab with soil from two maize-cropped agricultural fields. Microbial access to nutrients, including N, is at least partially determined by soil physical characteristics like bulk density (42). We leveraged two topographically distinct sites with differing soil properties, to assess how the comparability of field and lab measurements might vary in relation to soil characteristics. Field and lab procedures were kept as consistent as possible to determine the field relevance of lab qSIP measurements.

## MATERIALS AND METHODS

### Site description and experimental setup

We examined N cycling in two maize-cropped agricultural fields in Morgantown, West Virginia, USA, in August 2020. These fields were located at the West Virginia University Organic Research and Outreach Center (39.650910, –79.937906) and the West Virginia University Animal Science and Husbandry Research and Outreach Center (39.661446, –79.926243). The sites are in the same watershed, but topographically distinct. Hereafter, the WVU Organic Farm field will be referred to as the “Ridge” site and the WVU Animal Science farm field as the “Valley” site due to their distinguishing topographical characteristics. At each site, the experimental work was conducted in fields planted with maize. The Valley site is a footslope that has been under regular row crop production (mostly maize) for over a century. The Valley soil is classified as fine-loamy, mixed, superactive, mesic Oxyaquic Fragiudalfs in the Clarksburg series (43). Prior to maize planting, the field was cultivated with a triticale winter cover crop, which was mowed and baled in mid-May 2020, followed by the application of a broad-spectrum glyphosate-based herbicide. The Ridge site is a topographical shoulder that was previously naturalized grassland and was not in annual production until maize planting in June 2020. The Ridge soil is classified as fine-loamy, mixed, superactive to active, mesic Ultic Hapludalfs in the Westmoreland (WeC), and Dormont/Guernsey (DgC) soil series (43). At both sites, dairy manure and straw compost were applied and tilled into the soil a few days before maize planting in early June 2020. These sites were chosen for their distinct geomorphic positions, soil types, and land-usage histories to enable us to assess whether the field relevance of lab measurements is context-dependent.

In late July 2020, seven 1 m × 1 m plots were randomly established within a 10 m × 10 m area at each site (*n* = 7). Plots were centered around maize rows and gently hand-weeded as needed. For each plot, two 7.5 cm diameter PVC collars were placed approximately 4 cm deep within 15 cm of a maize stalk to prepare for the field qSIP measurements (Fig. S1a). Identical collars were also placed to measure N immobilization and mineralization. In addition, a 25 cm diameter PVC collar was installed within 15 cm of a maize stalk in each plot to measure field soil respiration.

Field and lab qSIP measurements were collected in mid-August 2020. At this time, the maize had reached the grain fill stage (V12–V15 stages), a stage commonly used for ^15^N pulse labeling experiments to determine nutrient availability for yield prediction (44). Before starting the incubations, soil cores were collected outside the collars to determine gravimetric soil moisture (45), water holding capacity (WHC) (46), and bulk density via the excavation method (47).

### Soil sampling and characterization

**Fig 1:**
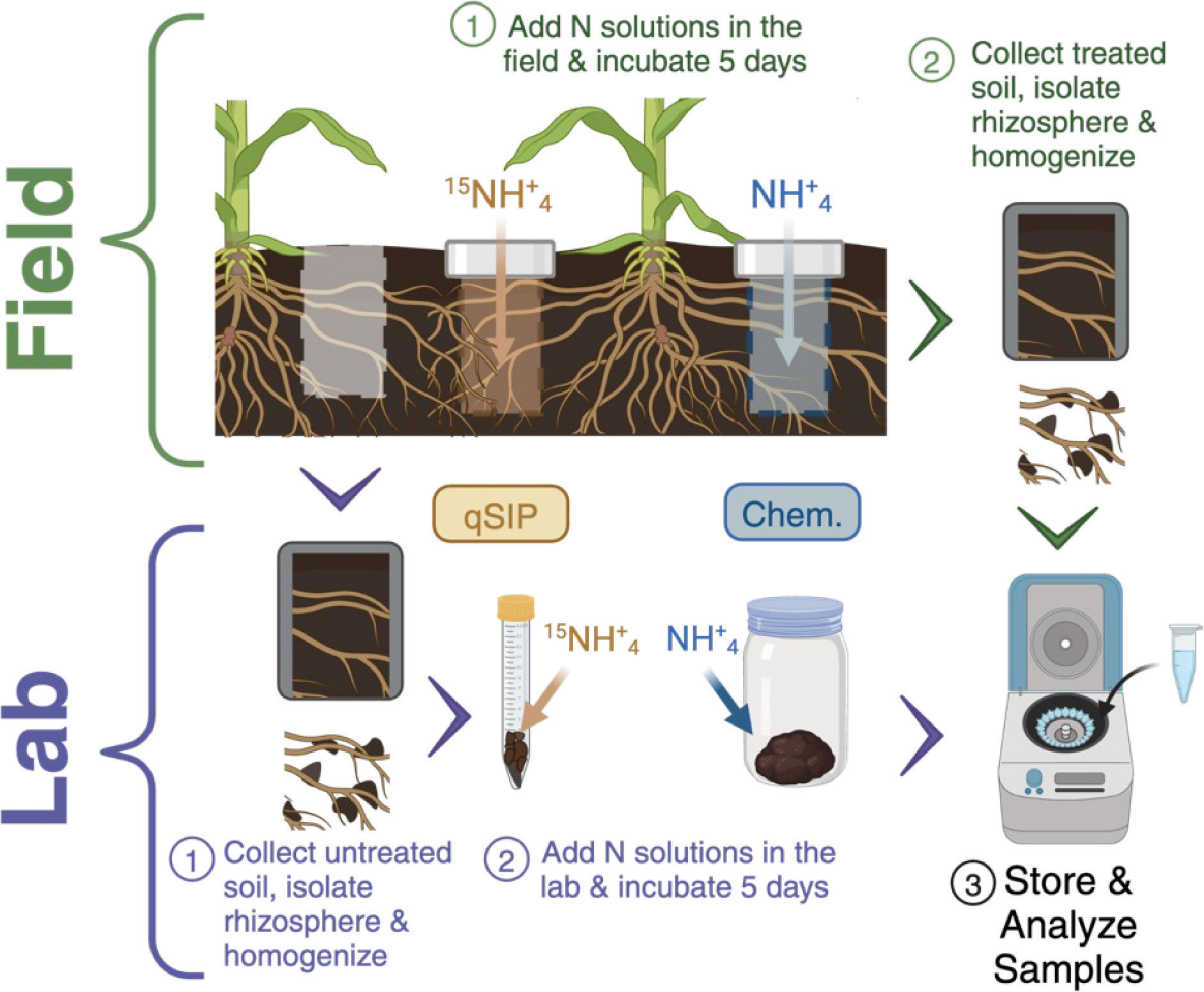
Graphical representation of tandem field and lab qSIP methods. The field samples were exposed to ^15^NH_4_ completely intact, whereas the lab samples were taken to the lab, where they were homogenized and sieved before ^15^NH_4_ addition. The same sample handling (except with NH_4_) was used for field and lab comparisons of soil biogeochemistry (“Chem.”) including N transformations, microbial biomass C, and CO_2_ flux. Created with BioRender.com.

To assess genus-specific ^15^N assimilation in the lab and field, simultaneous stable isotope incubations were performed (Fig. 1).

For the lab incubation, soil cores collected were within 15 cm of a maize stalk using a soil hammer corer (AMS 2 × 6 inch [5 × 15 cm] Soil Core Sampler). In total, over the length of the experiment, four soil cores were taken from each replicate plot and brought to the laboratory for separate analyses (Lab qSIP, Lab Chem., Field qSIP, and Field Chem., as shown in Fig. 1). Rhizosphere and bulk soil were separated by root picking and handshaking. Soil remaining on the root was classified as rhizosphere soil and the soil that fell off as bulk soil (48). Total C, N, and sulfur (S) of both bulk and rhizosphere soil were analyzed using the vario MAX cube (Elementar, Landenselbold, Germany). Soil pH was measured on bulk soil using an Accumet AE150 pH meter (Fisher Scientific, Hampton, NH, USA) (49). Soil organic matter (SOM) was determined on bulk soil *via* loss-on-ignition (50).

### Experimental incubations

Within 24 hours from the initial collection, rhizosphere soil was collected and sieved for initial community characterization and the lab qSIP incubation. For initial community characterization, 3 g of soil was subsampled and frozen at −80°C to represent the “pre-incubation” microbial community at natural abundance ^15^N (i.e., unlabeled) for both field and lab measurements. For the lab qSIP incubation, 3 g of the rhizosphere soil was incubated with 98 atom % (^15^NH_4_)_2_SO_4_ at a concentration of 3 µmole N g^−1^ soil at 60% WHC in Falcon tubes. All lab incubations were carried out in the dark at room temperature (~21°C). The amount of N added (3 µmole N g^−1^) is roughly equivalent to 83.3 kg N ha ^−1^, assuming a soil depth of 6 inches (15.24 cm) and a bulk density of 1.3 g/cm³, and is comparable to a late-season fertilization event (51).

The field qSIP incubation was initiated on the same day as the lab incubation, by adding 98 atom % (^15^NH_4_)_2_SO_4_ to achieve 3 µmole N g^−1^ soil (equivalent to ~42 µg natural abundance N g^−1^ soil). Before injection, pilot holes were made using 15.24 cm long, 3.2 mm diameter drill bits (Dewalt) to prevent soil compaction and clogging in the injection needle. The solution was injected into the soil within the PVC collar to a depth of 15 cm using a 50 mL syringe and 15.24 cm steel needle with 12 side ports (Hold Your Horses BBQ, Professional Meat Injection Kit). The solution was injected gradually throughout several pilot holes (similar to references 12, 41). The needle was slowly withdrawn and turned to achieve an even distribution of the solution throughout the soil column (Fig. S1b). The soil was fully saturated (100% water holding capacity) for an even distribution of (^15^NH_4_)_2_SO_4_. The PVC collars were loosely covered with a 10.2 cm PVC cap supported by rubber props to minimize leaching during rain events while allowing sufficient airflow.

In addition, a parallel lab and field incubation were set up to measure soil biogeochemistry (“Chem.” in Fig. 1) including CO_2_ flux and N transformation (see details below). Apart from unlabeled (^14^NH_4_)_2_SO_4_ addition (3 µmole N g Soil^−1^), the biogeochemical incubations were identical in conditions to qSIP field (100% WHC) and lab (60% WHC) incubation. The field soil was saturated initially to account for the loss of solution through evaporation and leaching over the incubation and to ensure adequate microbial isotope uptake, whereas the lab soil was saturated to 60% WHC but in an enclosed environment. Though field and lab soils initially had different moisture contents, we found that at the point of sample collection in the field the soil moisture had decreased from 100% to ~40% WHC (Table 2), suggesting that the field soil moisture probably experienced a similar WHC (60%) to the lab incubations over much of the incubation period. For the lab incubation, approximately 25 g of rhizosphere soil was incubated in a 960 mL mason jar fitted with a rubber septum for gas sampling.

Since inorganic N assimilation mainly occurs within the first week of fertilizer N addition, often peaking by the 3rd day (33), both lab and field incubations were halted after 5 days. No significant rainfall events occurred during this time and the surface soil temperature averaged around 22°C. Field qSIP cores were collected to a depth of 15 cm and transported back to the lab on ice. For both methods, soil samples were stored at −80°C.

### N immobilization and nitrification

Net nitrogen immobilization and nitrification were measured in the field and the lab under conditions identical to those used for qSIP. To measure initial N concentrations, an intact soil core (15 cm length × 5 cm diameter plastic sleeve) was transported to the lab in a sleeve and injected with a ^14^(NH_4_)_2_SO_4_ solution while still in the sleeve. After injection, rhizosphere soil was immediately collected, homogenized, sieved, and extracted for inorganic N analysis. Inorganic N was analyzed using soil subsamples collected upon initiation and completion of the 5-day incubation. Subsamples (8 g) were extracted with 40 mL of 0.5 M K_2_SO_4_ for 1 hour and stored at −20°C until analysis using an AQ300 Discrete Chemical Analyzer (SEAL Analytical, Mequon, WI, USA), following U.S. Environmental Protection Agency (EPA) methods 353.2 for nitrate and nitrite and 350.1 for ammonium (52). Net nitrification and immobilization rates were calculated as described by Hart et al (53).

### CO_2_ flux and microbial biomass carbon

Soil CO_2_ flux measurements were measured in the field at 0, 24, and 72 hours, over bare soil after initiation of the qSIP incubation using a LI-7810 Smart Chamber (LI-COR Biosciences, Lincoln, Nebraska). In the lab, cumulative soil respiration was measured using the closed chamber technique with a LI-6400XT as described in Walkup et al.(26). These measurements were used to calculate the average rate of C respiration in μg C kg soil^−1^ hour^−1^ during the 5-day incubation period. At the end of the incubation period, microbial biomass C was determined from 4 g subsamples of both lab and field soils, as described in Kane et al. (54).

### Plant biomass and ^15^N uptake

Upon termination of the 5-day incubation, the closest three maize plants to the field incubation cores were measured for height, and aboveground biomass was collected. The roots in the collected qSIP cores were rinsed, dried, and weighed before being stored at −80°C. Plant material was dried and then partitioned, as described in de Oliveira Silva (44), before being weighed and ground using a Wiley mill. The ground material was homogenized and subsequently analyzed for δ^15^N and %N using a Carlo-Erba NC 2500 Elemental Analyzer at the University of Maryland Central Appalachian Stable Isotope Facility. The rate of plant ^15^N uptake was calculated as follows: (atom% excess of sample  × total N)/(atom % excess of atmosphere  × incubation time) following the method outlined in Smercina et al. (55).

### Quantitative stable isotope probing

Total DNA extraction was conducted using the PowerLyzer PowerSoil DNA extraction kit, following the manufacturer’s instructions (MoBio Laboratories, Carlsbad, CA, USA). DNA quantification was performed using the Qubit dsDNA high-sensitivity assay kit and a Qubit 2.0 fluorometer (Invitrogen, Eugene, OR, USA). Further quantification of total DNA was performed via PicoGreen (Molecular Probe Inc., Eugene, OR, USA). To assess ^15^N uptake by microbial taxa, qSIP (7) was employed based on the methodology described by Morrissey et al. (15), with slight modifications described in Purcell et al. (56). For density centrifugation, approximately 2 µg of DNA was added to 3.5 mL of saturated CsCl solution (density of 1.89 g/mL). The 4.7 mL OptiSeal ultracentrifuge tube (Beckman Coulter, Fullerton, CA, USA) was then filled completely with a gradient buffer (200 mM Tris, 200 mM KCl, 2 mM EDTA). The samples were centrifuged in an Optima Max benchtop ultracentrifuge (Beckman Coulter) using a Beckman TLN-100 rotor at 55,000 rpm for 72 hours at 18°C. Subsequently, the density gradient was fractionated, and approximately 140 µL fractions were collected using a density gradient fractionation system (Brandel, Gaithersburg, MD, USA), resulting in approximately 25 fractions per sample. The density of each fraction was determined using a Reichert AR200 digital refractometer (Reichert Analytical Instruments, Depew, NY, USA). Contamination was avoided by thorough cleaning of the entire fractionation system and refractometer between tubes. Following density fractionation, DNA was purified from each fraction using isopropanol precipitation. Fractions near the ends of the density gradient in each tube had very low levels of DNA suggesting minimal contamination across fractions.

The quantification of the 16S rRNA gene in each fraction, from all samples, was determined using quantitative PCR (qPCR). All qPCRs were performed in triplicate and consisted of 2 µL of DNA template, 0.2 µM each of the 515F and 806R primer sets, 7.5 µL SYBR Green Master Mix (BIO-RAD, Hercules, CA, USA), and molecular grade water to a final volume of 15 µL per reaction. Thermal cycling conditions were as follows: 95°C for 2 min, followed by 40 cycles of 95°C for 30 s, 55°C for 30 s, and 72°C for 60 s. qPCR efficiencies were between 90% and 110%, the standard curve slope values were around −3.2 and R^2^ values were around 0.99. Based on the number of 16S rRNA gene copies in each fraction, we selected those fractions that had sufficient microbial DNA present in them for amplicon sequencing; these fractions had a density ranging from 1.67 to 1.74 g mL^−1^. Amplicon sequencing of the V4 hyper-variable region of the 16S rRNA gene was conducted using Illumina-compatible, dual-indexed 515f/806 primers, following the protocol by Kozich et al. (57). The Illumina MiSeq platform was used for sequencing, employing a 2 × 250 bp paired-end format at the Michigan State University Research Technology Support Facility Genomics Core.

### Data analysis

Sequence data were analyzed using MacQIIME2 (58), following the QIIME2 pipeline for sequence analysis, as previously described by Purcell et al. (56). In brief, the sequences were demultiplexed, joined (using vsearch), quality-filtered (with the removal of one sample), and denoised (depurated) using the Deblur algorithm with a trim length of 250 (59). To improve data quality, amplicon sequence variants (ASVs) occurring less than 200 times and in less than 20 fractions, across all samples, were removed. Taxonomy assignment to ASVs was performed using the q2-feature-classifier plugin with a pre-trained naive Bayes classifier for the 515F/806R 16S region of the SILVA database version 138, 99% identity ASVs (60). Downstream analyses were then performed using genus-level operational taxonomic units (OTUs), wherein each “genus” contained one or more ASVs with identical genus-level taxonomy (“*Level 6*” *in SILVA*). OTUs that did not belong to a described genus within the database and could not be classified at the genus level are referred to using the finest taxonomic level that could be assigned followed by “gen.” in the figures and throughout this text as an “unclassified” or “uncultured” genus in their respective taxonomic group (i.e., Family or Order). Relative abundance tables were exported for qSIP calculations, following the methodology of Finley et al. (8). An indicator species analysis, using the R package “indicspecies,” was conducted to determine which groups were different between the sites (61).

The “pre-incubation” samples were used to determine the natural abundance-weighted average density (WAD) and GC content of DNA as in Koch et al. (2018) and others (10, 12, 62). For each pair of ^15^N samples (unlabeled/pre-incubation and labeled/post-incubation) in each treatment (site and method type), the change in WAD of each genus was calculated. The WAD for each genus was calculated using the 16S rRNA gene copies (qPCR) for each genus in each fraction and weighted by the proportional abundance of total 16S rRNA gene copies measured in that fraction for each sample. Low-abundance taxa were filtered by setting a relative abundance threshold of less than 0.0001 (proportion), resulting in the removal of 11 genera. In addition, 179 taxa that were present in fewer than 5 samples per site (out of 7 total) were excluded. These filtering steps retained 98.6% of genera, all of which (511 total) were present in both the lab and the field. A tube correction was applied to account for tube-by-tube variation in WAD caused by variability in CsCl density, as described in Morrissey et al. (63), using the 50 genera with the smallest WAD shifts.

Following the approach described by Hungate et al. (7) with adjustments for ^15^N as outlined in Morrissey et al. (15), the excess atom fraction (EAF) of ^15^N incorporated into a genus’ DNA was determined based on the post-incubation shift in WAD. Negative values (estimated EAF ^15^*N* < 0) were replaced with zero for subsequent analyses. The ^15^N EAF reflects the amount of assimilated ^15^N in DNA, relative to the total N in DNA, over the incubation period (5 days) and is hereafter referred to as the “relative N assimilation rate” (similar to reference 64). The proportion of N assimilated by each genus “% N assimilated” was calculated as the product of each taxon’s relative abundance (expressed as a proportion) and ^15^N EAF divided by the sum of the product of relative abundance and ^15^N EAF for all genera as in (15).

To evaluate differences in ^15^N enrichment between methodologies, Equivariant Passing-Bablok Regression (65) was performed using the “mcr” package (66), following the methodology of Bablok (67) and Dufey (68). Passing-Bablok regression is a non-parametric and robust method commonly employed in method comparison studies (69, 70). It assumes a high correlation and linear relationship between the two variables being compared. Interpretation of Passing-Bablok regressions involves examining the confidence intervals for both the slope and intercept. If the confidence interval for the slope does not include the value of 1, it indicates statistically significant evidence of a proportional difference between the two methods. Similarly, if the confidence interval for the intercept does not include the value of 0, it suggests statistically significant evidence of a constant difference between the methods.

For visualization, we identified the 20 genera with the highest relative rates and total N assimilation values for each method (lab or field), averaged across both sites. Because top genera were not always shared between the two methods, more than 20 genera were reported. Ridgeline plots were created using the R package “ggridges” (71) and show the variation in taxon-specific measurements across the replicates. Differences in taxon-specific relative N assimilation rates or N assimilated (%) between the lab and the field methods were determined using one-way ANOVA (*n* = 7 per method).

Differences in soil characteristics and biogeochemical processes among sites, and where applicable, method, were assessed using one- and two-way ANOVA. Differences in prokaryotic community composition were assessed with two-way PerMANOVA using the R package “vegan” (72). Calculations, filtering, subsequent analyses, and figure creation were performed using R version 4.3.2 (73) in RStudio version 2024.4.0.735 (74).

## RESULTS

The Valley and Ridge soils differed in several physical and chemical characteristics (Table 1) despite their geographical proximity. The Valley site soil had significantly higher SOM (Welch t-test, *P* < 0.05), soil C and N contents, and a higher pH. The Valley site had a higher bulk density, and soil moisture in the field qSIP core after the incubation (*P* < 0.01), and tended to have higher soil WHC (*P* < 0.10). The Valley site also had shorter plants (*P* = 0.023) and tended to have lower plant biomass (*P* = 0.096) and plant uptake of the ^15^N-label (*P* = 0.097).

**TABLE 1:** Plant and soil properties at the Valley and Ridge sites (mean ± standard error)^*a*^

	Measurement	Valley	Ridge	*P*-value
Plant (*Zea mays*)	Biomass (g)	159 ± 17.5	211 ± 22.6	0.0958
	Height (m)	2.34 ± 0.06	2.53 ± 0.03	0.0228
	^15^ Nitrogen uptake (%)^*b*^	51.4 ± 6.60	70.4 ± 8.17	0.0967
Rhizosphere soil	Nitrogen (%)	0.42 ± 0.02	0.33 ± 0.01	0.0008
	Carbon (%)	3.98 ± 0.22	2.95 ± 0.16	0.0031
	Carbon:Nitrogen	9.40 ± 0.20	8.92 ± 0.15	0.0875
	Sulfur (%)	0.063 ± 0.002	0.062 ± 8e-04	0.0781
	Soil moisture (%)	24.0 ± 1.20	18.4 ± 1.00	0.0048
Bulk soil	Soil organic matter (%)	7.89 ± 0.34	6.49 ± 0.23	0.0059
	pH	5.83 ± 0.02	5.70 ± 0.03	0.0054
	Water holding capacity (%)	70.0 ± 1.63	65.8 ± 1.09	0.0562
	Bulk density (g/cm^3^)	1.34	1.13	

^*a*^ Significant difference (*P*-value < 0.05) between sites was determined via Welch two-sample *t*-test (*n* = 7 per site, except for bulk density where *n* = 1).

^*b*^ % of the total ^15^N isotope added to the soil.

### Biogeochemistry and microbial community

Carbon and N cycling process rates differed between field and lab methodologies (Table 2). Rates of CO_2_ flux were consistent at the Ridge site in field and lab measurements while at the Valley site, field-measured CO_2_ flux was 39% higher than lab-measured.

**TABLE 2:** Microbial processes measured in the field and lab at the Ridge and Valley sites (means ± SEM, *n* = 7 per treatment group)^*a*^

Parameter	Valley		Ridge		Two-way ANOVA
	Field	Lab	Field	Lab	
Net nitrification (μg N g soil^−1^ day^-1^)	0.80 ± 0.34	3.15 ± 0.35	3.57 ± 1.03	4.21 ± 0.75	Site, method
Net immobilization (μg N g soil^−1^ day^−1^)	0.675 ± 1.81^b^	2.25 ± 0.84^ab^	−2.16 ± 1.92^b^	6.94 ± 1.12^a^	Method, interaction
CO_2_ flux (μg C kg soil^−1^ hour^−1^)	27.5 ± 1.17^a^	16.8 ± 1.40^b^	26.7 ± 1.65^a^	25.3 ± 1.53^a^	Site, method, interaction
Microbial biomass C (mg C g soil^−1^)	3.35 ± 0.31	3.86 ± 0.60	3.52 ± 0.37	4.31 ± 0.41	Not significant

^*a*^ A two-way ANOVA was used to determine significant differences between sites and methods. When an interaction was present, Tukey HSD was performed and differences are indicated with lowercase letters (*⍺* = 0.05).

Rates of net nitrification and immobilization were higher when measured in the lab relative to the field (Table 2) and higher overall at the Ridge site than at the Valley site. Net immobilization rates did not significantly differ between sites. There was no significant effect of method or site on microbial biomass C.

Whole prokaryotic community composition was distinct between sites (PerMANOVA; F = 9.49, *P* = 0.001) and did not differ by method (F = 1.07, *P* = 0.281) (NMDS Fig. S2; 75). An indicator species analysis of total communities prior to the experiment (pre-incubation) showed the Valley site had significantly higher (*P* < 0.05) proportions of *Bacillaceae*, *Gaiellaceae*, *Nitrososphaeraceae*, *Blrii41*, and *Gemmatimonadaceae*, whereas the Ridge site had more *Xanthobacteraceae*, *Chtoniobacteraceae*, *Xiphinematobacteraceae*, *Vicinamibacteraceae*, and *Haliangiaceae*.

An indicator species analysis of the total communities from both sites by incubation method (field or lab) showed the field soils had more Firmicute families (including but not limited to *Thermoactinomycetaceae*, *Peptostreptococcaceae*, and *Bacillaceae*), Chloroflexi families, *Beijerinckiaceae* (free-living aerobic nitrogen-fixing bacteria in Pseudomonadota), *Nitrososphaeraceae*, and *Micromonosporaceae*. Lab soils had more Acidobacteriota, *Chitinophagaceae*, Methylomirabilota, Planctomycetota families (most notably *Isosphaeraceae*), *Rhizobiaceae*, and *Xanthobacteraceae*.

### Genus-specific nitrogen assimilation

**Fig 2:**
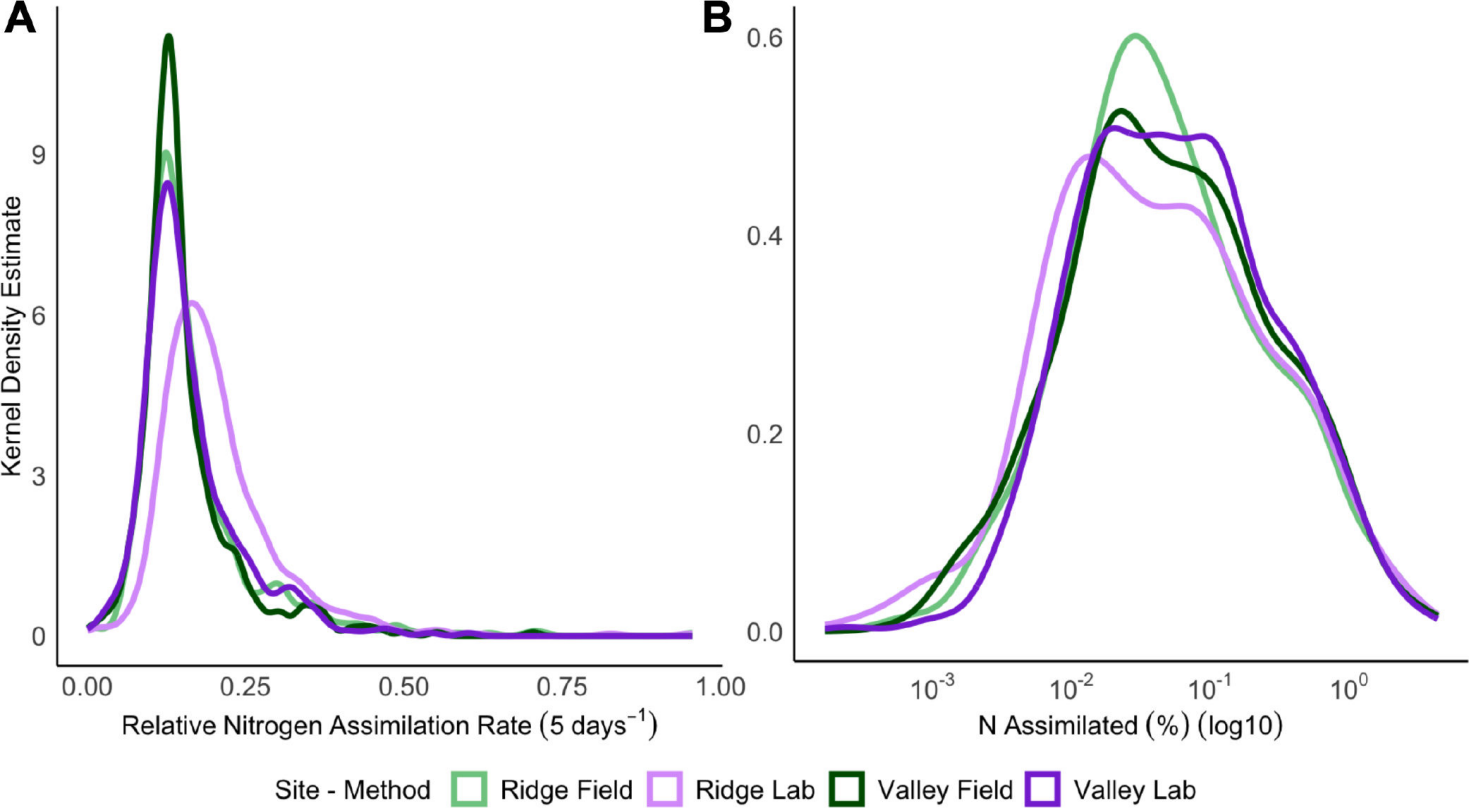
Kernel density estimate plots, which use a probability density function to create smoothed histograms, across all values of the taxon-specific (A) median relative N assimilation rates and (B) the median N assimilated (%) for all genera (note log_10_ scale). Relative N assimilation rate (i.e., excess atom fraction [EAF] ^15^N) reflects the amount of assimilated ^15^N in DNA, relative to the total N in DNA, over the incubation period. The % N assimilated estimates each taxon’s contribution to total N assimilation based on its EAF ^15^N and relative abundance.

Relative N assimilation rates were calculated for each genus and reflected the uptake of ^15^N into that organism’s DNA during the 5-day incubation period in the lab or field. The distribution of relative N assimilation rates for the Valley site was similar in the lab and the field (Fig. 2A). In the Ridge site, some taxa exhibited higher relative assimilation rates in the lab. When relative assimilation rates were weighted by relative abundance to estimate the proportion of N assimilated by each genus (% N assimilated), the distributions were highly similar across sites and methods (Fig. 2B).

**Fig 3:**
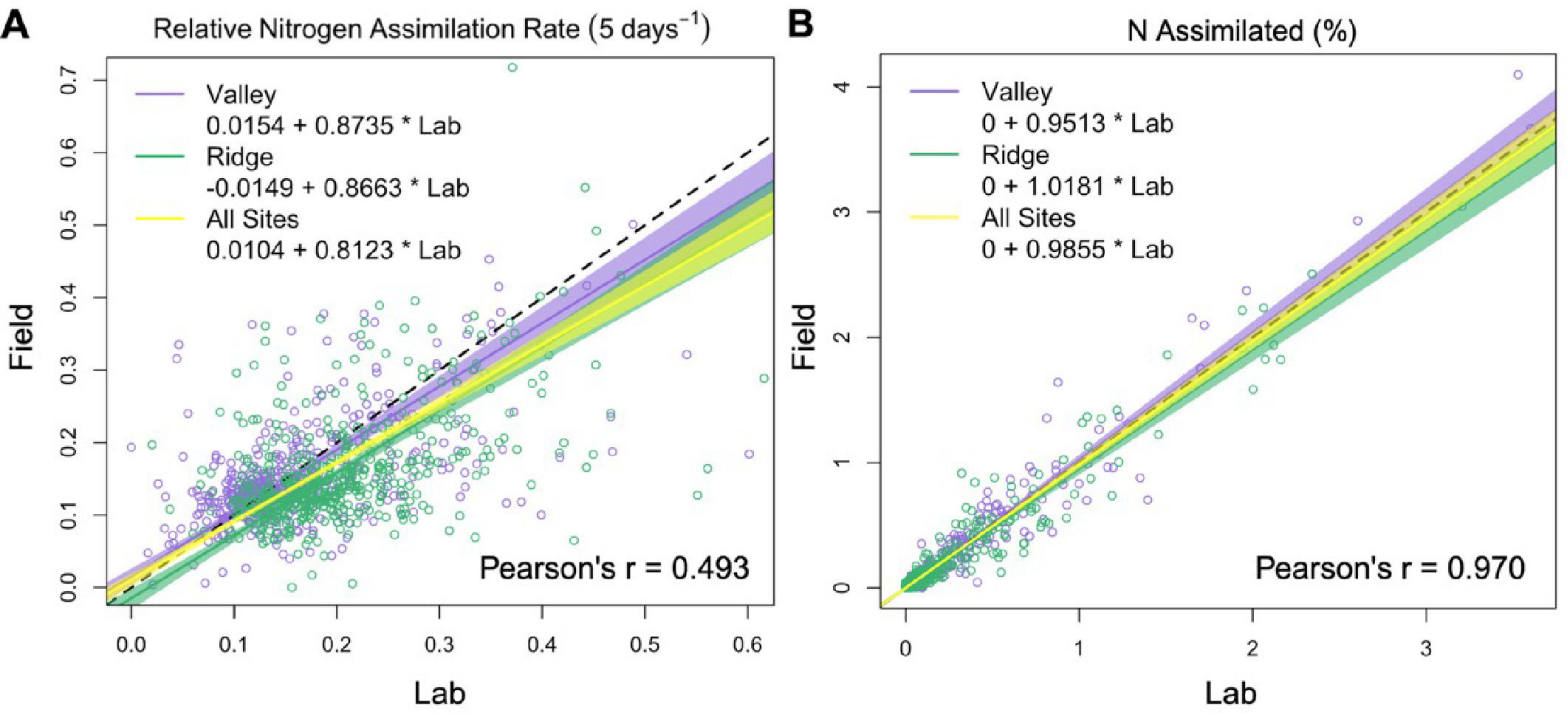
Equivariant Passing-Bablok regressions of median lab and field measurements of genus-specific (A) relative N assimilation rates and (B) the N assimilated (%) for each site independently (purple and green) and both sites together (yellow). Shading indicates the 95% confidence intervals calculated via the quantile bootstrap method. The black dashed line represents a 1:1 relationship. Pearson’s r was calculated using data from both sites.

The relationships between lab and field measurements of relative N assimilation rate and N assimilated (%) were assessed using an equivariant Passing-Bablok regression for each site independently, and both sites together (Fig. 3C and D). Similar regression results were obtained for the Valley and Ridge sites, so only combined values are discussed. For the relative N assimilation rate, field measurements covaried with those from the lab (Pearson’s r = 0.493; Fig. 3C); the Passing-Bablok regression slope was 0.81 (95% CI = 0.76 and 0.87) and the intercept was 0.01 (95% CI = 0.001 and 0.019). Since the confidence interval for the slope and the intercept did not overlap 1, this provides evidence of proportional and constant differences between methods, respectively. In this case, field measurements of the relative N assimilation rate were, in general, lower than those measured in the lab. The difference between field and lab methods largely disappeared when examining the proportion of N assimilated by each genus (Fig. 3D). Field and lab measurements were highly correlated across both sites (Pearson’s r = 0.97). In addition, the Passing-Bablok regression slope was 0.99 (95% CI = 0.95 and 1.02) and the intercept was 0.0000 (95% CI = −0.0001 and 0.000), indicating no proportional (slope) or constant (intercept) difference between the methods.

**Fig 4:**
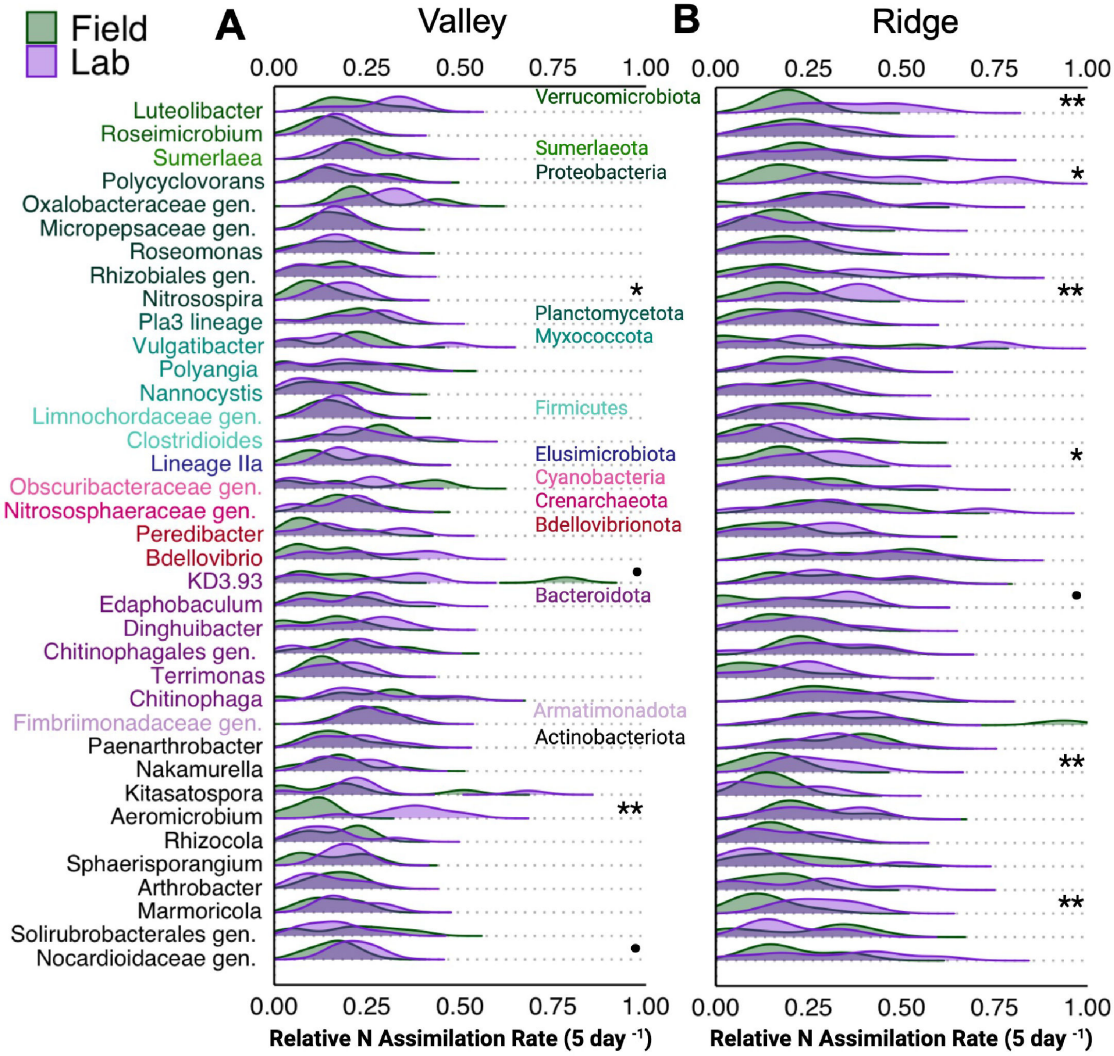
Ridgeline plots of the relative N assimilation rates for genera (y-axis) with the greatest median values for each method (Field and Lab) averaged across both sites (*n* = 7), not for each site individually. For ease of visualization, the (A) Valley and (B) Ridge sites are shown separately. Genera are grouped by Phylum. A Family or Order level classification followed by “gen.” refers to an unclassified or uncultured genus within the preceding taxonomic group. Significant differences (one-way ANOVA) between the field and lab are indicated as follows: •, *P* = 0.10; *, *P* = 0.05; **, *P* = 0.01; ***, *P* = 0.001.

**Fig 5:**
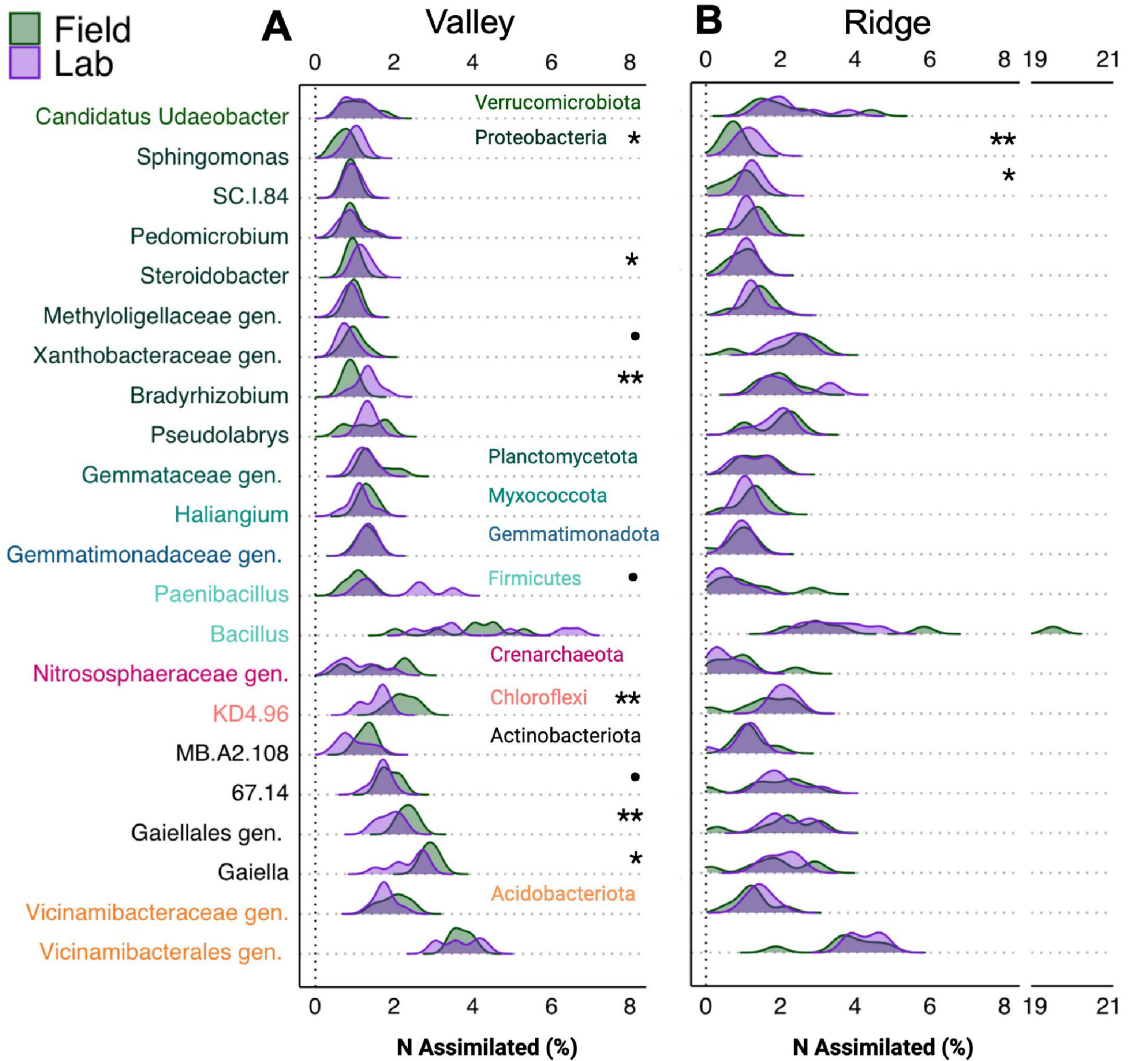
Ridgeline plots of the N assimilated (%) for genera (y-axis) with the greatest median values for each method (Field and Lab) averaged across sites (*n* = 7). For visualization, the (A) Valley and (B) Ridge sites are shown separately. Genera are grouped by Phylum. The Ridge (**B**) plot has a truncated axis, for visualization, to represent a field replicate for the genus Bacillus with a high value. Family or Order level classification followed by “gen.” refers to an unclassified or uncultured genus within the preceding taxonomic group. Note that significant differences (one-way ANOVA) between the field and lab are indicated as follows: •, *P* = 0.10; *, *P* = 0.05; **, *P* = 0.01; ***, *P* = 0.001.

Genera with relatively high median N assimilation rates across both sites occasionally differed between the lab and field (Fig. 4; Supp. Data). The genera responsible for the largest N assimilation rates were moderately consistent between methods; 17 genera of those reported were in the top 20 only in one method (Fig. 5; Supp Data). In the field, the highest relative N assimilation rates were observed for *Chitinophaga*, an unassigned genus within *Solirubrobacterales*, an unassigned genus within *Nitrososphaeraceae*, and *Sumerlaea* (Fig. 4; Supp Data). For lab measurements, the highest rates were observed for *Aeromicrobium*, an unknown genus within *Oxalobacteraceae*, *Luteolibacter*, *Bdellovibrio*, and *Chitinophaga*. Field measurements of relative N assimilation rates differed significantly from lab measurements at one or both sites for 7 of 37 genera with the highest relative N assimilation rates (~19% of top genera; one-way ANOVA *P* < 0.05). When considering all genera across both sites, the lab N assimilation rates were on average 16% higher than field rates. By site, lab assimilation rates were 23% higher for all genera at the Ridge site but only 7% higher at the Valley site. Among the top genera, only the genus *Nitrosospira* had significantly higher lab rates at both sites (Ridge + 53%, *P* = 0.005; Valley + 39%, *P* = 0.02). At the Valley site, *Aeromicrobium* (Valley + 69%, *P* = 0.002) was clearly higher in the lab, but an unassigned genus within *Nocardioidaceae* (Valley + 22%, *P* = 0.05) and *Bdellovibrio* (Valley + 61%, *P* = 0.07) also tended to have higher lab rates. The Ridge site had more taxa with higher lab rates including *Luteolibacter* (Ridge + 44%, *P* = 0.007), *Lineage Ila* (Ridge + 42%, *P* = 0.03), *Nakamurella* (Ridge + 43%, *P* = 0.009), *Marmoricola* (Ridge + 56%, *P* = 0.006), and *Polycyclovorans* (Ridge + 60%, *P* = 0.01). *Edaphobaculum* also had slightly higher lab rates (Ridge + 48%, *P* = 0.10).

Genera with relatively high % N assimilation across both sites differed regularly between the lab and field methods only at the Valley site (Fig. 5; Supp. Data). The genera responsible for the largest proportions of N assimilated were consistent across both methods; only 2 of the reported genera were in the top 20 only in one method (Fig. 5; Supp Data). The top five were an uncultured *Vicinamibacterales* genus, *Bacillus*, *Gaiella*, an uncultured *Gaiellales* genus, and *KD4.96*. Field measurements of the proportion of the N assimilated differed from lab measurements for 7 of the top 22 genera (~32%) at one or both sites (one-way ANOVA; *P* < 0.05). Of the top assimilators, only *Sphingomonas*’ % N assimilation was significantly different at both sites, being higher in the lab (Valley + 33%, *P* = 0.02; Ridge + 38%, *P* = 0.005). Uniquely at the Ridge site, only *SC.I.84* differed between methods and was higher in the lab (+18%, *P* = 0.03). At the Valley site, several genera were higher in the lab including *Bradyrhizobium* (+35%, *P* = 0.003), *Steroidobacter* (+17%, *P* = 0.01), and *Paenibacillus* (+45%, *P* = 0.09), while others were higher in the field including *Gaiella* (+11% Field, *P* = 0.02), *KD4.96* (+23%, Valley *P* = 0.001), an uncultured *Gaiellales genus* (+17%, *P* = 0.006), *67.14* (+3%, *P* = 0.09), *MB.A2.108* (+40%, *P* = 0.10), and an uncultured *Xanthobacteraceae* genus (+26%, *P* = 0.10).

At the phylum level, the relative N assimilation rates and proportions of N assimilated were generally consistent between the field and the lab (Fig. S3A). Similar to the genera level results, the mean relative N assimilation rate at the phylum level across both sites was ~15% higher in the lab compared to the field. By site, the mean rates were only 4% higher at the Valley site but 24% higher at the Ridge site.

## DISCUSSION

The development of qSIP was a methodological advance that permits the measurement of genus-specific rates of element assimilation (7), which can help open the “black box” of microbial ecology to advance our mechanistic understanding of below-ground ecosystem function. We aimed to build upon this to assess the comparability of lab-based qSIP measurements from agricultural soil with tandem field measurements (Fig. 1; Fig. S1). Overall, we found that relative N assimilation rates were generally lower in the field for the total community, but the magnitude of this difference varied by site and genera (Fig. 2A, 3A, and 4; Fig. S3A). The field and lab methods became much more comparable when the relative assimilation rates were weighted by relative abundance to estimate the proportion of N assimilated by each genus (% N assimilated), though this was also site- and genus-dependent (Fig. 2B, 3B, and 5; Fig. S3B). We identified several taxa that showed both high relative rates and % N assimilation across sites and methods, suggesting or confirming their importance for N retention and cycling in maize-cropped rhizosphere soils.

The novel methodological approach for field qSIP with ^15^N described here enabled the measurement of genus-specific relative N assimilation rates in relatively undisturbed rhizosphere soil. There were detectable levels of DNA enrichment with ^15^N in the field (Fig. 2A and B), alleviating concerns that plant competition for N, soil heterogeneity limiting access to the isotope solution, and leaching would prevent microorganisms from sufficiently assimilating the isotope. Overall, the field enrichment levels were broadly comparable to those measured in the lab for most taxa (Fig. 3A and B; Fig. S3), suggesting that lab qSIP methods provide a reasonable estimate of microbial activity in the field. Notably, dominant assimilators were phylogenetically diverse regardless of method (Fig. 4 and 5; Fig. S3). Compared to previous field qSIP experiments (11, 13, 14, 32, 41), our study differed in several important ways. First, we implemented a concurrent lab incubation for direct methods comparison. Second, the field qSIP incubation was conducted in the presence of live plants. Third, this experiment was conducted with agricultural soils. Fourth, we used (^15^NH_4_)_2_SO_4_ to investigate N assimilation specifically, instead of ^18^O water which is used to track growth rates more broadly. Due to the lower cost of ^15^N, we were able to use a much larger volume of soil, including more roots and capturing a larger spatial scale.

Lab and field measurements of ^15^N assimilation were correlated (Fig. 3), indicating that these lab measurements can help us generally understand microbial function in the wild. Although the correlation between the lab and field relative N assimilation rates was only of intermediate strength (Pearson’s r = 0.49), the rates did not differ between methods for 81% of high N assimilating taxa (Fig. 4). Most bacteria experience only a tiny local environment (76), as such laboratory conditions may have been sufficiently similar to field conditions to produce moderately similar N assimilation rates despite the disturbance associated with soil collection, sieving, and the absence of roots. Many genera also had similar relative N assimilation rates across both sites, suggesting some consistency in genus-specific microbial traits across environmental variation (77, 78).

The relative N assimilation rate enables the evaluation of a genus’ N assimilation ability, regardless of its abundance in the soil. This is valuable as it allows the detection of rarer taxa that influence soil nutrient acquisition processes (62, 79–81). However, abundance in soil must still be considered as a genus’ contribution to community-level processes scales in proportion to its relative abundance (10, 82). In our study, 31%–36% of added N was assimilated by the most abundant 5% of taxa. The % N assimilation is a relative abundance-weighted assessment of a genus’ contribution to N assimilation. When considering the % ^15^N assimilated by each genus, there was no significant difference between lab and field measurements overall, as indicated by the high correlation between these measures (Pearson’s r = 0.97, Fig. 3B). The similarity in community composition between the field and the lab ( Fig. S2) likely contributed to the agreement between lab and field measures of genus-specific % ^15^N assimilation. One limitation of comparing genus-specific % ^15^N assimilation values is that a genus’ relative abundance does not necessarily equate to biomass. Across all environments, prokaryotic cells can vary dramatically in size, and therefore biomass, typically between 0.2 and 5 µm in diameter, with the largest reaching 750 µm (83, 84). Therefore, this metric will likely overestimate the % ^15^N assimilation of smaller, more quickly replicating microbes, and underestimate it for larger, slow-growing microbes. Cross-referencing both relative N assimilation rates and the abundance-weighted % N assimilation can lead to useful inferences about the relevance of a particular genus’s N immobilization capabilities.

Despite some similarity between the lab and the field, relative N assimilation rates were generally lower in the field (Fig. 2A, 3A, and 4A). Interestingly, rates of net N immobilization and nitrification were also lower in the field (Table 2). Our results suggest this is primarily due to in-field plant uptake of ^15^N, reducing ^15^N availability to microbes. We found that ~50%–70% of the added ^15^N was recovered in plant biomass (Table 1). Field N assimilation may also have been lower due to loss of ^15^N through leaching or denitrification, though this is expected to be minimal for ammonium-based fertilizers (33). Soil physical heterogeneity could also have limited prokaryotic mobility and access to nutrients. However, this is unlikely as total microbial activity, measured through soil respiration, was either the same, or slightly higher when measured in the field (Table 2). Higher field respiration could be due to live-root respiration, fresh root C inputs, or bioturbation (85, 86). Given these respiration results, it seems likely that the reduced field N assimilation rates (Fig. 3A and 4A) and N cycling rates were due to N removal from the soil either by plants or leaching.

Soil characteristics and plant activity may impact the disparity between field and lab measurements. The Ridge soil had lower bulk density, water-holding capacity, and end-point soil moisture (Table 1), likely from lower clay and silt content compared to the Valley site’s bottomland soil (43). We can reasonably infer that the Ridge soil was less able to “hold onto” the isotope solution during the field incubation. This inference is also supported by our measurements of plant ^15^N uptake; ~70% of the added ^15^N at the Ridge site was utilized by the maize, compared to only ~51% at the Valley site (Table 1). This property of the Ridge soil likely led to the various site-specific differences in our analysis. The difference between field and lab method values was often larger at the Ridge site than at the Valley site (Fig. 2 and 4). Microbial community composition was less homogeneous at the Ridge site (Fig. 5; Fig. S2). A few taxa, most notably *Bacillus,* were highly abundant in several field qSIP samples at the Ridge site, reaching up to 19% of the total % N assimilated despite below-average relative assimilation rates (Fig. 5). *Bacillus* species can rapidly form robust biofilms on plant roots, including maize (87, 88). Wu et al. (89) found that fertilization induced soil biofilm formation and those biofilms sustained 40 times more active microbes than free-living cells (89). Planctomycetota can also be biofilm formers (90) and were found to have higher % N assimilation in the field (Fig. S3). Through the inclusion of live plant roots and maintenance of soil structure, it is possible that the field qSIP method captured this key process in some replicate samples.

The most effective microbial allies for N retention within agricultural soils are likely to be microbes that are both abundant and effective N assimilators. Within the top relative N assimilators, we identified three taxa in maize-rhizosphere soils with the highest % N assimilation and relative N assimilation: an unassigned genus in *Nitrososphaeraceae* (Crenarchaeota; 28%, 0.22), *Luteolibacter* (Verrucomicrobiota; 30%, 0.23), and *Terrimonas* (Bacteroidota; 22%, 0.15) (Fig. 4 and 5; Supp. Data). Of the taxa with the highest % N assimilation values (Fig. 5; Supp, Data), the only genus with a median relative assimilation rate above 0.14 was *Candidatus Udaeobacter* (Verrucomicrobiota; 1.43%, 0.149). A genus from the order *Vicinamibacterales* (Acidobacteriota) also had a very high % N assimilation (3.9%) and slightly above average relative N assimilation rate (0.13).

Despite some taxa being more active in either the field or lab, many active taxa could be identified using either method. Notably, *Chitinophaga* had some of the highest relative assimilation rates across both methods (91). For relative N assimilation rates, ~19% of the top assimilating genera had significantly different activities between methods, usually at only one site (Fig. 4). Only *Nitrospira* clearly had higher lab rates at both sites. *Nitrospira* are well known for their nitrifying capabilities, yet our results suggest they are also effective at N assimilation, indicating a potential dual role where they contribute both to soil N retention and loss (92). Many *Nitrospira* are considered opportunists (93). They likely benefited from the enhanced access to the N solution in sieved and mixed soil samples. For % N assimilation values, only 10% had significantly different activities between methods, all at only one site with the exception of *Sphingomonas*, which had higher lab % N assimilation at both sites (Fig. 5). Some *Sphingomonas* are opportunists, reliably taking advantage of soil disturbances and to N fertilization (94).

The lab method had higher relative N assimilation rates for most taxa (Fig. 2 and 3), but a few had higher N assimilation values in the field method: Gaiellales, Solirubrobacterales, Rhizobiales, Crenarchaeota, Chloroflexi, Planctomycetota, Armatimonadota, and Firmicutes (Fig. 4 and 5; Fig. S2 and S3; Supp. Data). Most of these taxa have been observed living in close association with, or within, plant roots (91, 95). Members of Rhizobiales and Xanthobacteraceae (also slightly higher % N assimilation at the Valley site) include diazotrophic taxa that live in, on, and around plant roots. Actinobacteria (Gaiellales, *67.14*, and *MB.A2.108,* Solirubrobacterales) are filamentous and some have mycelial structures spread throughout the soil (96). These lifestyle strategies are intimately tied to roots (e.g., Rhizobiales, Xanthobacteraceae) or soil structure (Gaiellales, Solirubrobacterales) and the removal of plant roots and disruption of soil structure may have led to their reduced uptake and/or abundance in the lab method.

Based on these results, the choice of qSIP methodology (field or lab) depends on the research objective. Field measurements may be most appropriate for researchers aiming to collect field-relevant data for downstream applications (climate models, precision fertilization, etc.) (97, 98). Capturing the realized, *in situ* rate of N assimilation and growth for soil microbes may be especially important for field experiments that manipulate environmental factors (e.g., fertilization, warming, (41)). Field measurements are likely to best capture the influence of plant-microbe interactions on the activities of rhizosphere symbionts (40, 99). Taxa with greater N assimilation in the field may form close associations, and N recycling networks, with maize roots and/or root-associated fungi (100, 101). Future field qSIP experiments should include fungal and other eukaryotic microbes, given the importance of these groups for N and C cycling in soil (22, 32, 33). Lab measurements, without root presence, may significantly undervalue the activity of these taxa in rhizosphere nutrient cycling in the field. However, the ability to carefully control experimental conditions is a clear advantage of lab measurements, and this approach may be preferable for measuring how microbial traits respond to environmental drivers (12). In addition, as lab measurements yielded higher genus-specific activity rates (Fig. 2 and 3), this approach may be preferable for answering questions regarding trait variation across microbial phylogeny where the influence of evolutionary history is most clear when growth or assimilation rates are comparatively high (77, 102). Even so, qSIP is predicted to have relatively high sensitivity and balanced accuracy, even at the lower levels of isotope incorporation seen in the field (103).

Well-replicated field qSIP captures the complex ecology of soil microbes, their environment, and their interactions where those microbes live and grow. Our results show that field qSIP with ^15^N can be used to measure N assimilation of microbial taxa in intact soil with living roots. While microbial relative N assimilation rates and their contribution to N cycling (% N assimilated) were contextually comparable in the lab and field, we did observe constant and proportional differences by method, the magnitude of which varied by site (Fig. 3). The lower relative rates of nitrogen assimilation observed in the field, mainly due to plant competition (Table 1), underscore the critical role of field conditions in shaping microbial activity. Field measurements may be preferred to accurately assess the activity of microbes that are heavily influenced by soil disturbance or the presence of living roots.

## Data Availability

Sequence data are available in the NCBI Sequence Read Archive under BioProject ID PRJNA1194143 (103). All original and derived data and code used are available at https://github.com/kinseyreed/2020_FieldqSIP (104). The processed data used to generate the figures and perform the analyses are available on Figshare (DOI: 10.6084/m9.figshare.27040441 (105).

## SUPPLEMENTAL MATERIAL

Supplemental figures aem.01849-24-s0001.docx Figures S1 to S3.

ASM does not own the copyrights to Supplemental Material that may be linked to, or accessed through, an article. The authors have granted ASM a non-exclusive, world-wide license to publish the Supplemental Material files. Please contact the corresponding author directly for reuse.
