# Supplementary material for: Comparing field and lab quantitative stable isotope probing for nitrogen assimilation in soil microbes
Source: Appl Environ Microbiol. 2025 Jan 16;91(2):e01849-24. doi: 10.1128/aem.01849-24 (PMC11837507; doi:10.1128/aem.01849-24)
Supplement: Supplemental figures — Figures S1 to S3. [file aem.01849-24-s0001.docx]

**Supplemental Figures for:**

Comparing field and lab quantitative stable isotope probing for nitrogen assimilation in soil microbes

Kinsey Reed, Chansotheary Dang, Jeth Walkup, Alicia Purcell, Bruce Hungate, Ember Morrissey

*Applied and Environmental Microbiology*

**A**
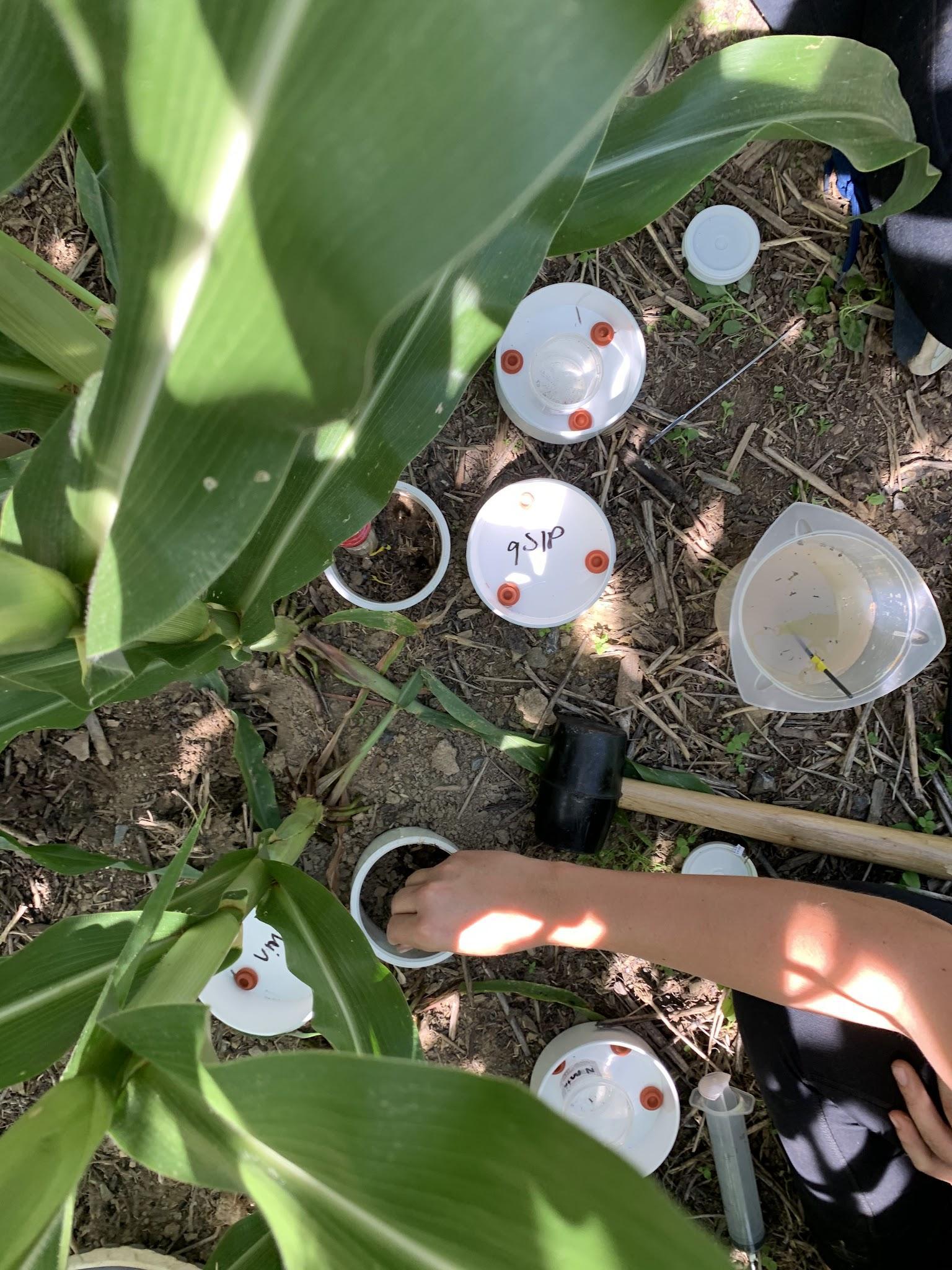
**B**
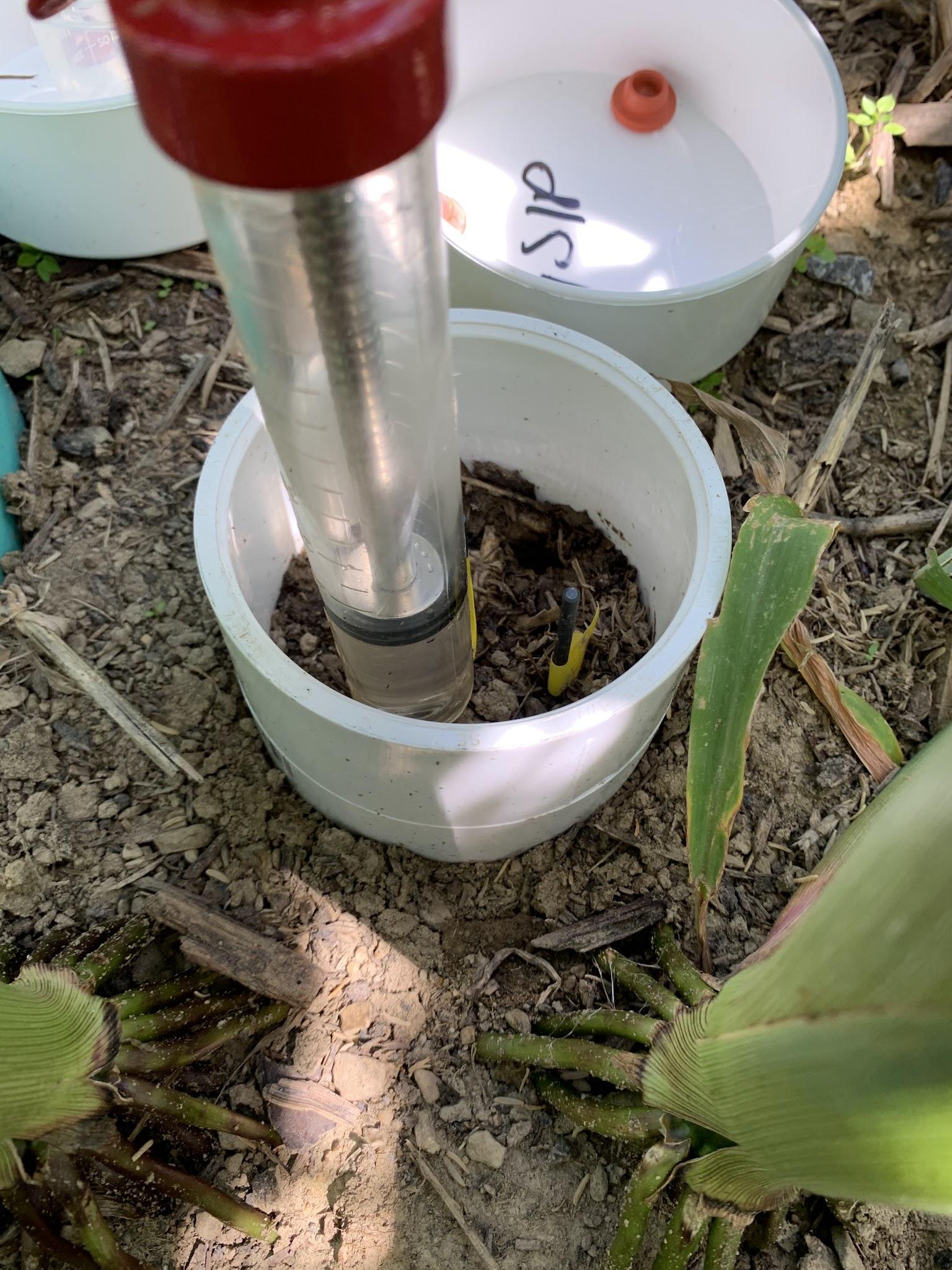


**Supplementary Figure 1.** Photographs of PVC collar placement for field qSIP (A), and isotope solution injection (B).


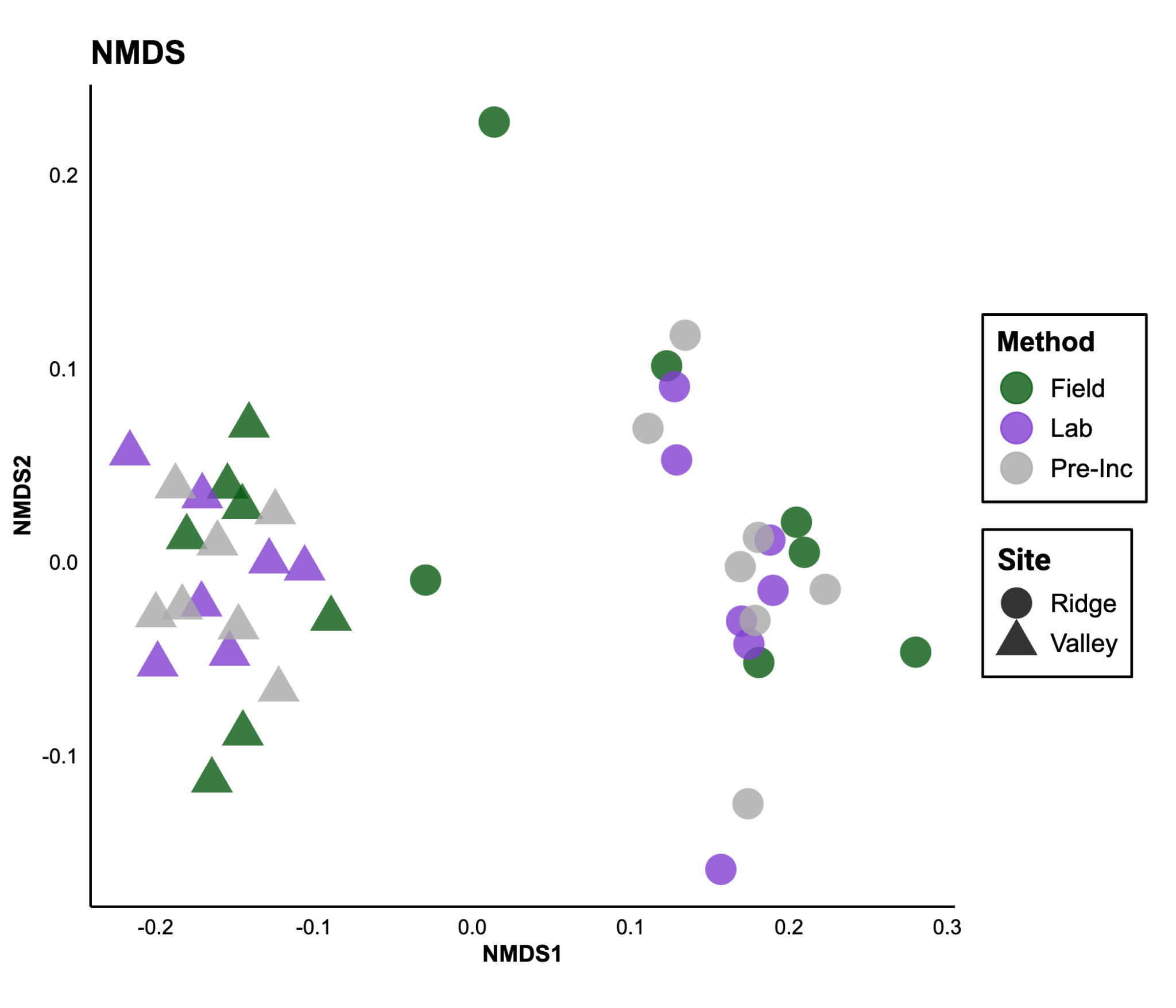
**Supplemental Figure 2.** Non-metric Multidimensional Scaling (NMDS) ordination of prokaryotic community composition (Bray-Curtis dissimilarity of 16S rRNA gene sequences) pre- and post-incubation. Grey colored shapes represent communities prior to incubation with ^15^NH_4_ in the field or lab.


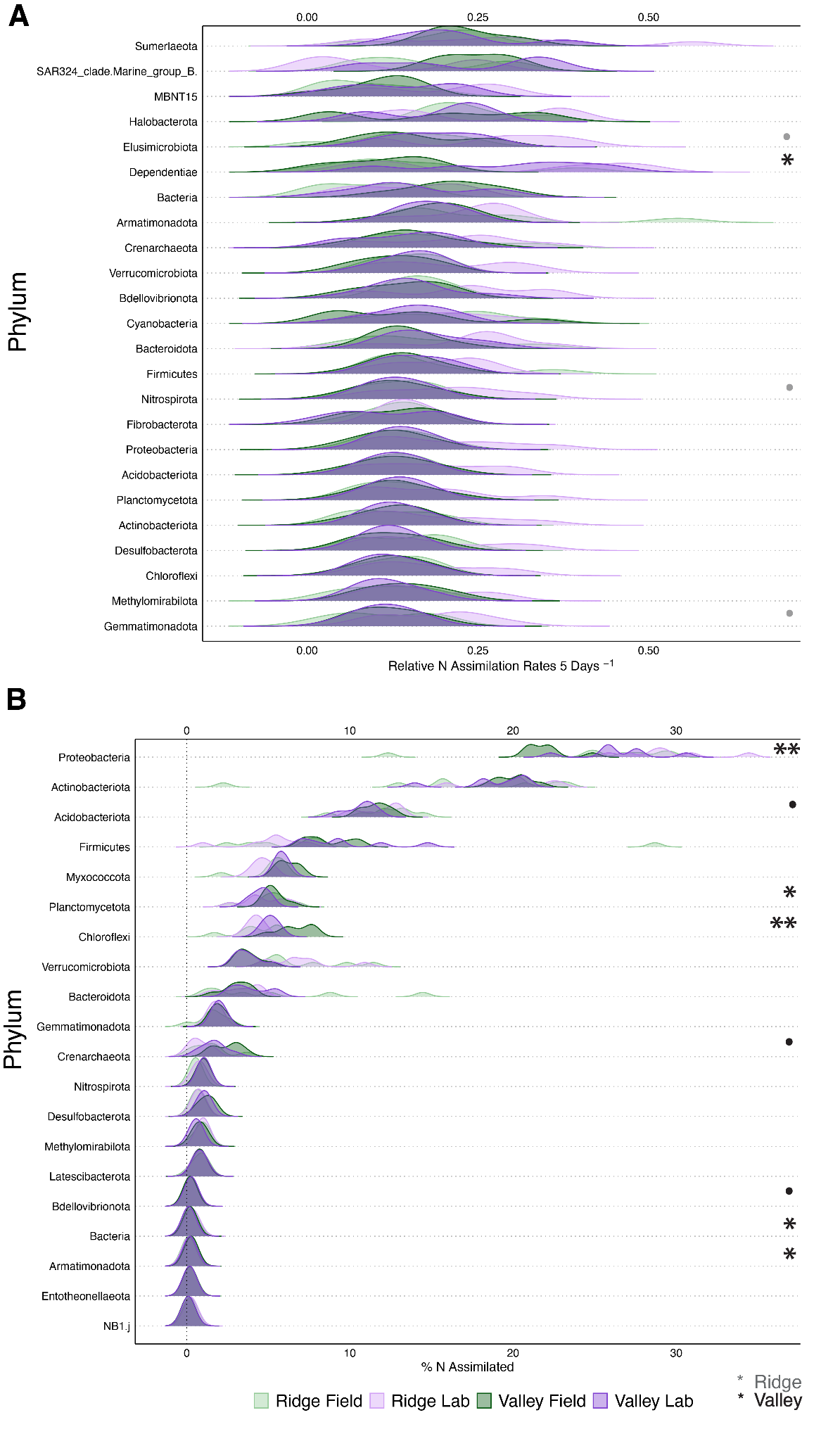


**Supplemental Fig 3.** Ridgeline plots of A) relative ^15^N assimilation rate over 5 days and B) % ^15^N assimilated (abundance-weighted) for phyla with the greatest median values for each method. Plots are ordered by highest median value across both sites and methods. Significant method effects (1-way ANOVA) for each phylum indicated with **^.^** = 0.10, * = 0.05, ** = 0.01 and colored by site.
